# Assessing Value in Child Health

**DOI:** 10.3390/children8110972

**Published:** 2021-10-27

**Authors:** Wendy J. Ungar

**Affiliations:** 1 Technology Assessment at Sick Kids (TASK), Child Health Evaluative Sciences, The Hospital for Sick Children (SickKids) Research Institute, Toronto, ON M5G 1X8, Canada; wendy.ungar@sickkids.ca; 2 Institute of Health Policy, Management and Evaluation, University of Toronto, Toronto, ON M5T 3M6, Canada

Assessing value in child health is increasingly important as health care systems face difficult choices with regard to what services and programs for children to fund and deliver. Measuring cost-effectiveness, evaluating effects on quality of life for patients and their caregivers, determining preferences for treatments and services from patients, families, and members of the public, and consideration of the social, legal, ethical, and equity implications of new complex technologies all contribute to an understanding of value. Conducting research to measure elements of value often necessitates novel methods and approaches. Conducting such research in child health is particularly challenging given the paucity of valid pediatric patient-reported outcome instruments, the lack of rigorous effectiveness data sources, reliance on proxy reporting, and many other methodologic and data-related constraints. 

This Special Issue features papers exploring topics that expand our understanding of value in child health. Not surprisingly, many papers focus on the measurement of health-related quality of life (HRQoL) [1,2,3,4]. In Lamb et al.’s review of how HRQoL was measured and reported for children under the age of 5 years in appraisals performed by the UK National Institute for Health and Care Excellence (NICE), telling challenges were revealed [3]. These included the use of adult instruments such as the EQ-5D-3L. The NICE appraisals acknowledged the “exceptional challenges” in valuing health states for very young children and infants. Based on their review, Lamb et al. proposed recommendations for research in pediatric HRQoL that have been taken up by other contributors to this Special Issue. In Petrou et al.’s study of HRQoL in adolescents with permanent childhood hearing loss, generic and preference-based instruments were compared for self and parent proxy reporters [4]. The research team found that the magnitude of decrement in HRQoL observed due to hearing loss depended on the tariff set applied to the Health Utility Index (HUI) Mark 2 and 3, as well as the choice of the reporter. The divergence between self and parent proxy reports has been previously reported, particularly for subjective or difficult-to-observe attributes of HRQoL, such as cognition and emotion [5]. Interestingly, Petrou et al. observed that parents reported lower scores for both subjective and objective elements of HRQoL and recommended reporting utilities from children and parent proxies as complementary, rather than interchangeable. With the aim of improving the HRQoL instrument choices available to researchers, Bashir et al. assessed the validity and reliability of a newer preference-based instrument, the Child Health Utility (CHU) 9D, through comparisons to HUI2 and HUI3 in children with inflammatory bowel disease [1]. CHU-9D utilities obtained from children with Crohn’s disease differed depending on whether an adolescent or adult tariff set was used. Generally, CHU9D utilities were lower than HUI2 and HUI3 with correlations between 0.62 to 0.69 between the various instruments and tariff sets, reinforcing the lack of interchangeability between tools. The need to fully understand the psychometric properties of available pediatric preference-based HRQoL measures is further expounded in Jones et al.’s multi-instrument comparison (P-MIC) study protocol. P-MIC will compare pediatric HRQoL instruments in 6100 Australian children and adolescents aged 2–18 years [2]. The instruments that will be studied include the CHU9D, Pediatric Quality of Life Inventory (PedsQL) Core 4.0, EQ-5D-Y, the Toddler and Infant Questionnaire (TANDI), EQ-5D-5L, HUI2/3, AQol-6D, Patient-Reported Outcome Measurement Information System 25 (PROMIS-25), KIDSCREEN-27, and disease-specific measures. The results of this ongoing study, expected in December 2022, should help provide guidance on optimal approaches to preference-based HRQoL measurement in children of various age groups. 

Understanding and accurately measuring HRQoL is but one aspect of full economic evaluation. A significant challenge that has received little attention is how to convert deficits in child cognitive function into a measurable future productivity loss that can be modeled in economic evaluations of interventions aimed at improving cognitive function over the lifespan. Using published estimates of the correlation between intelligence quotient (IQ) and market productivity, Gosse and Zhou valued a one-point improvement in IQ in the United States at USD 10,600–13,100 [6]. Using this estimate in economic evaluation underscores the importance of considering productivity gains or losses as a child grows to adulthood and the need to take a societal payer perspective so these changes can be measured and reported. 

Lifetime time horizons are essential for economic evaluations of precision medicine screening and diagnostic testing interventions that identify genetic or other molecular-based conditions that require lifelong treatment. Molecular biomarker tests and genome-sequencing technologies offer the ability to identify conditions early in childhood to facilitate treatment that may avert significant morbidity and mortality in the long term. It is not always clear, however, how to position these tests in an assessment pathway. Chen et al.’s study examined the incremental cost-effectiveness of identifying phenylketonuria, a genetic disorder, via newborn screening with a genetic test, compared with a symptom-based clinical diagnosis over the lifetime from a societal payer perspective [7]. Their model incorporated a comparison of alternative treatment regimens for positive cases consisting of diet or medication The finding that newborn screening with diet had an incremental cost-effectiveness ratio of USD 6400 per QALY gained compared with clinical identification with diet is important evidence to guide precision medicine policies for this population. Assessing the value of precision medicine technologies, in particular genome-sequencing strategies that deliver primary and secondary findings for patients and family members, has introduced numerous new challenges to the conduct of economic evaluations. Cernat et al. reviewed the literature to identify methodological challenges associated with incorporating cascade effects of genetic testing, i.e., effects in family members when a child receives a positive result [8]. Challenges related to study design, costing, and measurement and valuation of health outcomes were delineated, and recommended approaches were presented. Finally, given the significant implications of genetic testing in children for family members, Hayeems et al.’s work acknowledged that the concept of value must extend well beyond the traditional measurement of costs and health outcomes [9]. Their review identified elements of parents’ personal utility of genetic testing that were related to affective, cognitive, behavioral, social, and medical management domains. This understanding can enhance our ability to determine value for emerging genetic testing technologies for funding, policy, and clinical decision making.

This collection touches on critically important concepts in understanding value in child health and should inspire researchers and decision makers alike to think more broadly when considering how to value interventions and services that benefit children and their families. 

## Data Availability

Not applicable.
